# Examining the underlying latent structure of the sports emotion questionnaire: Insights from the bifactor multidimensional item response theory

**DOI:** 10.3389/fpsyg.2022.1038217

**Published:** 2022-12-22

**Authors:** John Elvis Hagan, Frank Quansah, Francis Ankomah, Edmond Kwesi Agormedah, Medina Srem-Sai, Thomas Schack

**Affiliations:** ^1^Department of Health, Physical Education and Recreation, University of Cape Coast, Cape Coast, Ghana; ^2^Neurocognition and Action-Biomechanics-Research Group, Faculty of Psychology and, Sports Science, Bielefeld University, Bielefeld, Germany; ^3^Department of Educational Foundations, University of Education, Winneba, Ghana; ^4^Department of Education and Psychology, University of Cape Coast, Cape Coast, Ghana; ^5^Department of Education, SDA College of Education, Asokore-Koforidua, Ghana; ^6^Department of Business and Social Sciences Education, University of Cape Coast, Coast, Ghana; ^7^Department of Health, Physical Education, Recreation and Sports, University of Education, Winneba, Ghana

**Keywords:** athletes, bifactor models, confirmatory models, cross-culture, emotions

## Abstract

**Background:**

Despite the widespread use of the sports emotion questionnaire (SEQ) in several studies, it is surprising that only a few have explicitly tested the validity and utility of the instrument in non-western populations. Besides, the issue of dimensionality and the latent structure of the instrument remain inconclusive given that several authors have revealed different factor structures across diverse populations. The central concern is whether the items on the various dimensions, proposed for the original SEQ, offer adequate information to their respective expected subscale or otherwise. This study assessed the underlying latent structure of the SEQ using confirmatory and bifactor multidimensional item response (MIRT) models.

**Methods:**

Through a well-designed validation study 300 athletes from three West African countries, participating in the 2018 West African University Games were surveyed to respond to the SEQ. The data were analyzed using first, a 5-factor confirmatory factor analysis (CFA) via the MIRT model and second, a bifactor MIRT analysis.

**Results:**

The results revealed that items on the SEQ were fairly good in measuring the construct under the respective domains of the instrument. However, the outcome of the bifactor model showed that the majority of the items on the SEQ explained common variance in relation to the general factor other than the specific domains (5-dimensions).

**Conclusion:**

Findings of the bifactor model question whether the sub-dimensions of the SEQ are needed since most of the items on the SEQ explained larger variances in the general factor than any of the five domains. It is concluded that instruments like SEQ should be scored for a general factor and not as sub-dimensions. Further investigations are encouraged by scholars within the area to probe the dimensionality of the SEQ.

## Introduction

Emotions have become a popular research topic within the field of sport psychology. This is due to the effects they can have on psychological-related processes and sports performance (e.g., Hanin, 2000; Lazarus, 2000; Jones and Uphill, 2011). Previous researchers have discovered that athletes/sport performers experience multitudinous emotions including happiness, excitement, relief, pride, anger, anxiety, dejection, guilt and shame in sport settings (e.g., Jackson, 2000; Raglin and Hanin, 2000; Hanin, 2007; Martinent et al., 2012) leading to sport performance variability (e.g., Lazarus, 2000; Hanin, 2007). Athletes’ pleasant/positive and unpleasant/negative emotions have the potential to either facilitate or impact sport performance depending on their dimensions (i.e., intensity, direction and frequency, e.g., Lazarus, 2000; Ruiz and Hanin, 2004; Hanin, 2007; Martinent and Ferrand, 2009). Research so far has examined the prevalence of emotions and relationships between different dimensions of emotions and performance, including coping using well-calibrated measures (e.g., Martinent et al., 2012; Latinjak et al., 2013; Arnold and Fletcher, 2015).

The sport emotion questionnaire (SEQ) emerged as one inventory that has gained prominence in evaluating a broad spectrum of emotions experienced during competition across different athletes from a multi-dimensional perspective (Jones et al., 2005), despite the existence of several other unidimensional scales like Sport Anxiety Scale (SAS; Smith et al., 1990), Competitive State Anxiety Inventory-2 (CSAI-2; Martens et al., 1990) and Competitive Aggressiveness and Anger Scale (CAAS; Maxwell and Moores, 2007). The SEQ was developed and validated considering several theories. For example, the authors drew from Fredrickson’s (2001) broaden-and-build theory to define the concept of emotion, Lane and Terry’s (2000) conceptual model to distinguish between emotion, and Lazarus and Folkman’s (1984) mood and affect, and appraisal theories of emotion to provide a foundation for understanding how discrete emotions are differentiated. The SEQ was developed to assess the emotions of individual athletes prior to competition. The SEQ assesses five emotions, grouped into two higher-order dimensions: positive emotions (excitement and happiness) and negative emotions (anxiety, dejection, and anger). The scale contains 22 items scored on a 5-point Likert scale from 0 = “not at all” to 4 = “extremely” where higher values indicate greater emotional intensity. The SEQ has been reported to have excellent reliability for its scales, with Cronbach’s alpha estimates ranging from 0.81 (excitement) to 0.88 (happiness). In addition, support has been provided for the factorial validity of the SEQ [Comparative Fit Index (CFI) = 0.93; Root Mean Square Error of Approximation (RMSEA) = 0.07] (Jones et al., 2005). Since SEQ’s development and validation, it has also been adopted by scholars to evaluate recalled emotions in a sport setting (e.g., Vast et al., 2010).

Although the SEQ has been in existence for over one decade, few studies have assessed its psychometric properties and performance across different geographical contexts such as in Spain (Latinjak et al., 2013; González-García et al., 2020), UK (Arnold and Fletcher, 2015), Turkey (Bayköse and Şakar, 2018), Germany (Wetzel et al., 2020), Persia (Vaez-Mosavi and Eshghi, 2020), and Portugal (Gomes et al., 2021). For example, González-García et al. (2020) using confirmatory factor analysis (CFA) discovered a five-factor model for SEQ in a Spanish version. The reliability co-efficient of the Spanish version of SEQ ranges from 0.77 (excitement) to 0.91 apiece (happiness and dejection), with acceptable measurement model fit indices (χ^2^ = 2720.15, *p* < 0.001, CFI = 0.90, RMSEA = 0.05). In Portugal, Gomes et al. (2021) adapted the SEQ to measure how referees felt about their next game. The study supported the five-factor model and the CFA revealed good psychometric properties for the instrument [χ^2^ (197) = 618.742, *p* < 0.001; CMIN/DF = 3.141; RMSEA = 0.074, 90% CI (0.067;0.080); CFI = 0.938, TLI = 0.928]. Vaez-Mosavi and Eshghi (2020) also ascertained the adaptability, validity and reliability of the Persian Version of the SEQ among athletes with different physical activity levels. Results of CFA, Cronbach’s alpha and intra-class correlation coefficients showed that the instrument supported the 5-factor structure with 22 items and adequate construct validity. Also, the reliability of the Persian version of SEQ was confirmed as highly valid.

Despite these confirmatory findings, other validation investigations produced contradictory results. For example, Arnold and Fletcher (2015) in the UK, tested the factor structure of the SEQ at a different time point (viz. the past month). Fit indices from the CFA provided partial support for the hypothesized measurement model, with equal or better fit revealed than evident in the initial validation. The measurement model fit indices were sufficient and above the acceptable guidelines for all factors at the subscale level. Also, in Germany, Wetzel et al. (2020) validated the English SEQ. The authors found that a factor structure deviating from the original SEQ was found, whereupon SEQ-d (a name proposed by the Wetzel et al. to depict a shorter-version of the SEQ) was developed as a three-dimensional short scale. The SEQ-d showed acceptable fit indices (CFI = 0.950, RMSEA = 0.069, SRMR = 0.063) and the internal consistency was α = 0.84 (negative emotions), α = 0.86 (positive emotions), α = 0.87 (tension). Similarly, Bayköse and Şakar (2018) assessed the construct validity of SEQ in Turkish culture using 191 athletes via Exploratory Factor Analysis (EFA) and CFA. Instead of a five-factor, the authors found a four-factor structure after the adaptation process to Turkish culture. The goodness of fit parameters (X^2^/*df* = 2.370, CFI: 0.95, SRMR: 0.003, RMSA: 0.08) concluded that the Turkish form of SEQ was adequately valid and reliable.

A critical and relevant issue regarding the ongoing calibration of SEQ is the issue of dimensionality and the latent structure of the instrument. The inconsistencies in the factor structure of SEQ in several contexts present a concern to scholars using the instrument (Latinjak et al., 2013; Arnold and Fletcher, 2015; Bayköse and Şakar, 2018; Wetzel et al., 2020). The central concern is whether the items on the various dimensions, proposed for the original SEQ, offer adequate information to their respective expected subscale since previous validation studies have found that some items offered very little information to their dimension (Latinjak et al., 2013; Bayköse and Şakar, 2018). Studies that have assessed the dimensionality of SEQ usually adopted statistical procedures (e.g., EFA and CFA) which only examine whether or not an item provides much information about a particular sub-domain of the construct. With previous studies relying predominantly on CFA, a theory-based specification is fitted that necessitates every item to be assigned to a single factor. The challenge with this approach is that there is no information on the percentage of variances an item contributes to the general trait (i.e., general emotions). Thus, there has been less attention paid to instances where a particular item can provide information for the general emotion construct as well as information to a sub-domain, which a bifactor multidimensional item response theory (MIRT) would help provide such information (Reckase, 2009; Liang et al., 2021).

Bifactor models assess the simultaneous influence of a general factor and specific factors on a set of indicators with the assumption that the specific factors are uncorrelated since the shared variance among the specific factors is a result of the general factor (Reise, 2012; DeMars, 2013; Flores-Kanter et al., 2018). In the case of the SEQ, for example, an item fixed under the happiness sub-domain may not only provide information for that sub-scale but could offer significant information for the measurement of general factor emotions. Meanwhile, previous studies have shown a strong relationship between indicators of happiness and other constructs like excitement, dejection and anger (see Vast et al., 2010). It is not surprising, therefore, to hypothesize that the totality of the specific items can be influenced by the same latent construct; comprised of specific factors that are linked to a general factor like emotions (Dominguez-Lara and Rodriguez, 2017). Questions about whether responses from the SEQ should be summed up to obtain a general score for analysis, or whether responses should be composed into sub-domains instead remain unanswered. This study attempts to address this question through 5-factor confirmatory and bifactor MIRT models.

Further, despite the documented evidence of emotional encounters among African professional athletes (e.g., Hagan et al., 2017a,b; Hagan, 2021; Srem-Sai et al., 2021a), it is surprising that no research has assessed the validity of the SEQ using these cohorts in the African context. This is a significant gap in the literature that needs earnest consideration, particularly when the SEQ has been employed by several investigators in sport psychology research across different boundaries (e.g., Britton et al., 2019; Hagan, 2021; Ruiz et al., 2021; Srem-Sai et al., 2021a). Findings from previous validation inquiries (e.g., Latinjak et al., 2013; Arnold and Fletcher, 2015; Bayköse and Şakar, 2018; González-García et al., 2020; Vaez-Mosavi and Eshghi, 2020; Wetzel et al., 2020; Gomes et al., 2021) may produce fluctuating applicability results in different settings, for example, Africa given the collectivist idea of its setting rather than the individualistic idea of Western countries (e.g., Spain: Latinjak et al., 2013; González-García et al., 2020), (UK: Arnold and Fletcher, 2015), (Turkey: Bayköse and Şakar, 2018), (Germany: Wetzel et al., 2020) and some Asia countries (e.g., Persia: Vaez-Mosavi and Eshghi, 2020) where cultural, social beliefs and practices may likewise fluctuate. Sport performers’ diverse shared norms, social behaviors, and values influence their emotional experiences during sport competition (Hagan, 2021). Thus, respondents’ understanding and interpretation of the items of SEQ in African settings like Ghana might vary from those of different nationalities in the western world (Srem-Sai et al., 2021b; Quansah et al., 2022a). Consequently, SEQ’s materiality and applicability in other geographical boundaries may not be certain.

To date, the psychometric properties and performance of the SEQ in the African context are still lacking. In professional football, where emotions encountered among players are common, adapting the SEQ in an African environment could provide valuable information on emotional evaluation situations for appropriate coping interventions. Additionally, discrepancies in previous validation studies’ findings (e.g., Latinjak et al., 2013; Arnold and Fletcher, 2015; Bayköse and Şakar, 2018; González-García et al., 2020; Vaez-Mosavi and Eshghi, 2020; Wetzel et al., 2020; Gomes et al., 2021) suggest that more research is needed to determine the quality and applicability of the SEQ using different samples (e.g., non-Western respondents). This study assessed the underlying latent structure of the SEQ using confirmatory and bifactor MIRT models. Particularly, the study tested (1) how the data on SEQ from African athletes fit the 5-factor confirmatory MIRT model, (2) how the data on SEQ from African athletes is consistent with the proposed bifactor MIRT model, and (3) how the bifactor MIRT model compares with the 5-factor confirmatory MIRT model based on the data on SEQ from African athletes.

## Materials and methods

### Research design

A validation study was adopted as the research design. This research design was appropriate because this study went through distinct scientific stages from the planning phase through to the estimation of sample size, data collection and the evaluation of the reliability and validity with different statistical tools (Arafat, 2016a). Although there happens to be predominant use of a cross-sectional survey design in recent validation studies (see Agormedah et al., 2022; Ankomah et al., 2022; Quansah et al., 2022b,c; Srem-Sai et al., 2022), emerging knowledge from scholars is that validation study can be considered as a research design since every stage of validation research has scientifically established rudiments that contradicts the fundamental idea of cross-sectional design (Anthoine et al., 2014; Arafat, 2016b; Arafat et al., 2016).

### Participants’ selection and information

A sample of 300 student-athletes (164 males and 136 females) from Benin (*n* = 54), Nigeria (*n* = 150), and Ghana (*n* = 96) were selected during the 2018 West Africa University Games (WAUG) held in Nigeria using a convenience sampling technique. The sample size selected was guided by the recommendations of Linacre (1994) who through simulation studies endorsed a sample limit of 300 to ensure stability across the item parameters. Participants were aged between 19 and 34 years (*M* = 25.95 years, *SD* = 3.259) where the number of Muslims, Christians and those in other religions like Buddhists, African Traditional religion and Hinduists were 87, 177 and 36, respectively. The competitive statuses of participants involved being either a regional athlete (*n* = 31), a national athlete (*n* = 125) and/or an international athlete (*n* = 144).

Different public universities in the various African sub-regions had formally admitted these student-athletes to study various academic programs at the graduate and undergraduate levels for the award of several degrees and certificates. To qualify as a regional athlete, the participant must have competed consistently for their home countries and won several awards at the regional or district levels or both. Moreover, a student-athlete would qualify as a national athlete, only if the individual had participated and won several awards at the national level within their home country whilst qualifying as an international athlete required participants to have competed at the international level for their home country at different times, won several awards nationally and involved in various African regional competitions (Hanton et al., 2005). The sporting events that participants competed in during the 2018 WAUG games in Nigeria were athletics (*n* = 150) handball (*n* = 24), volleyball (*n* = 24), basketball (*n* = 24) and football (*n* = 78). Coaches, delegation leaders and team captains were reached at their residing hostels and hotels for assistance in recruiting the study sample at the competition venue. Every competitor was optimistic and hoped to win several awards for their beloved countries.

### Instrumentation

#### Sport emotion questionnaire (SEQ)

The 22-item SEQ, developed by Jones et al. (2005) was employed to measure the pre-competition emotions of the study participants. The items are classified into five (5) subscales, namely dejection (5 items: *disappointed, sad, unhappy, dejected* and *upset*); anxiety (5 items: *tense, nervous, anxious, uneasy* and *apprehensive*); anger (4 items: *angry, annoyed, furious* and *irritated*); happiness (4 items: *happy, joyful, cheerful* and *pleased*) and excitement (4 items: *energetic, enthusiastic, excited* and *exhilarated*). Happiness and excitement are further classified as positive or pleasant emotions whilst dejection, anxiety and anger are classified as negative or unpleasant emotions. Every item is scored on a 5-point Likert type scale, ranging from 0 (*not at all*), 1 (*a little*), 2 (*moderately*), 3 (*quite a bit*) and 4 (*extremely*). Participants were asked to indicate how they were feeling “right now, at this moment, regarding the upcoming competition” within the competitive setting in the week prior to the actual competition. The psychometric properties of the SEQ have been established using face, content, factorial, and concurrent validity tests. Moreover, Cronbach alpha reliability coefficients ranging from 0.74 to 0.90 have been reported previously for the SEQ.

### Procedure and quality control measures

Following compliance and adherence to all ethical standards, this validation research procedure was officially endorsed by the Institutional Review Board (IRB) at Bielefeld University, Germany. An additional endorsement was sought from accompanying officials and leaders of delegations from the three African countries (i.e., Benin, Ghana and Nigeria) at the WAUG 2018 games. The study was conducted following the Declaration of Helsinki (Bošnjak, 2001; World Medical Association, 2001; Tyebkhan, 2003), which sets out the fundamental ethical principles for research involving human subjects. Additionally, the research was conducted in compliance with the Standards of Ethics in Sport and Exercise Science Research (Harriss et al., 2017).

Participants were asked to sign written informed consent forms before the data collection started. Direct recruitment of the participants began after the researchers and their assistants enquired from coaches and captains of all teams from the three African countries whilst at the same time good rapport was being established. Participants were all informed not to write their names on the survey instrument for the sake of anonymity. They were also assured that every information provided would be kept confidential and further informed that their willingness or otherwise to continue and/or complete responding to the survey items at any point they deemed necessary was voluntary without any consequences. Before administering the SEQ to participants, the standard instructions for the instrument were thoroughly explained to the participants to enhance clarity without ambiguities. The meanings of each item on the questionnaire (i.e., SEQ) were explained regarding the emotions they felt in the week before the WAUG competitions.

Two research assistants who were fluent in English and French, and could also speak some of the indigenous local dialects within the three countries were employed to assist and facilitate the researchers in the data collection process. The research assistants helped to distribute the instrument with pencils to the study sample to answer. While the majority of the participants responded to the instrument immediately, others requested that the research assistants pick theirs up before the opening ceremony. Answering the questionnaires took about 30 min for each respondent. Just before the opening ceremony, the research assistants collected all the answered questionnaires and sealed them at the participants’ hostels.

### Data analyses

The descriptive statistics of the items on the SEQ were presented. These include the mean, number of responses, standard deviation, skewness and kurtosis. Based on the 5-factor structure of the SEQ, two different models were fitted using the *mirt* package^1^ in the *R*-environment (Chalmers, 2012). A 5-factor CFA was first performed via the MIRT model, followed by a bifactor MIRT analysis (Reckase, 2009). To judge the adequacy of each model, the following fit indices with their associated criteria were used: Tucker-Lewis Index (value should be greater than 0.90); Goodness-of-Fit Index (value should be greater than 0.90); CFI (should be greater than 0.90); Standardized Root Mean Square Residual (should be less than 0.08); and RMSEA (should be less than 0.10) (Kline, 2015). Other indicators were also used to decide which model was optimal: Akaike Information Criterion (AIC), Bayesian Information Criterion (BIC) and Sample-adjusted BIC (SABIC) and Log-likelihood (LL). Models with lower values on the information indicators were considered as optimal (Kass and Wasserman, 1995). Discrimination parameters were assessed based on the recommendations of Reckase and McKinley (1991) that estimates below 0.50 are poor, 0.50–1.0 is moderate and an index greater than 1.0 shows good to excellent discrimination). The threshold parameter represented the capability of items to distinguish between respondents with different levels of emotion. A threshold step criteria of 0.81 was utilized for assessing the functioning of the response categories (Wolfe and Smith, 2007). This threshold step cut-off was derived from a simulation study which showed that a minimum threshold step of 0.81 is sufficient to suggest that the response categories of these thresholds do not overlap.

The model used for the 5-factor CFA model and the bifactor MIRT model is the graded response model which assumes that there are unique response categories for each item with a particular intercept (Samejima, 1969). The parameter estimation procedure for the bifactor model was the fixed quadrature expectation-maximization whereas the Metropolis-Hastings Robbins-Monro estimation method was used for the 5-factor CFA model (Chalmers, 2012). For the bifactor model, it was assumed that the specific factors are uncorrelated. Also, the 5-factor CFA and bifactor models were nested and this allowed for the chi-square difference test to be used for comparison. Additional analysis was performed using the “*bifactorIndicesCalculator”* package in R software to further conclude on the appropriate model to use (Dueber, 2020). First, four different omega coefficients were computed, namely, Total Omega (ω), Subscale Omega (ω_*s*_), Hierarchical Omega (ω_*H*_), and Hierarchical Omega for subscales (ω_*HS*_). The omega estimate computes the amount of variance in the composite observed scores that can be accounted for by all common sources of variances (McDonald, 2013; Reise et al., 2013). Other bifactor indices estimated include the Explained Common Variance (ECV), Relative Omega, Percentage of Uncontaminated Correlations (PUC) (i.e., entire model), Factor Determinacy (FD), and Construct Replicability (H).

For interpretation purposes, values of ω_*H*_ greater than 0.80 reflect unidimensional measure which by extension helps to explain the values of ω_*HS*_ indicating the percentage of systematic variations of scores from a specific domain after segregating the variances accounted for by the general factor (Reise et al., 2013). The ECV for the general factor is the amount of all common variances accounted for by that factor. The ECV for the specific domains reflects the strength of a sub-domain relative to all explained variances of all the items, including items not loading on that domain of interest (Stucky and Edelen, 2015). There are instances where ECV for specific factors can be computed to include only items loading on the specific domain, which is called ECV (NEW). The *H-*measure explains the relationship between a specific factor and an optimally weighted item composite where a high *H* estimate, usually greater than 0.80, indicates a well-defined construct (Mueller and Hancock, 2008). The *FD* measure represents the multiple relationships between the scores from an item and the factor with estimates greater than 0.90 preferred (Grice, 2001). The PUC estimate describes the proportion of covariance terms that solely explain the general factor (Rodriguez et al., 2015). ECV and PUC values greater than 0.70 suggest that the scale should be treated as if it was unidimensional, however, when PUC and ECV estimates are less than 0.80 and 0.60, respectively, the scale should be treated as multidimensional (Reise et al., 2013; Rodriguez et al., 2015).

## Results

### Descriptive analyses

The descriptive statistics of the items on the SEQ are presented in Table 1.

**TABLE 1:** Mean, standard deviation, skewness and kurtosis of the SEQ items.

SN	Items	N	Mean	SD	Skewness		Kurtosis	
					Statistic	Std. error	Statistic	Std. error
Q1	Uneasy	300	1.657	1.383	0.405	0.141	–1.048	0.281
Q2	Upset	300	1.443	1.329	0.351	0.141	–1.298	0.281
Q3	Exhilarated	300	1.883	1.427	0.054	0.141	–1.285	0.281
Q4	Irritated	300	1.737	1.371	0.037	0.141	–1.287	0.281
Q5	Pleased	300	2.063	1.224	–0.166	0.141	–0.832	0.281
Q6	Tense	300	1.633	1.282	0.280	0.141	–1.012	0.281
Q7	Sad	300	1.693	1.461	0.200	0.141	–1.363	0.281
Q8	Excited	300	2.040	1.418	–0.014	0.141	–1.292	0.281
Q9	Furious	300	1.993	1.468	0.031	0.141	–1.347	0.281
Q10	Joyful	300	2.090	1.410	–0.060	0.141	–1.282	0.281
Q11	Nervous	300	1.873	1.370	0.144	0.141	–1.144	0.281
Q12	Unhappy	300	1.633	1.449	0.227	0.141	–1.310	0.281
Q13	Enthusiastic	300	1.820	1.362	0.017	0.141	–1.198	0.281
Q14	Annoyed	300	1.780	1.383	0.118	0.141	–1.251	0.281
Q15	Cheerful	300	1.957	1.447	0.023	0.141	–1.277	0.281
Q16	Apprehensive	300	1.613	1.425	0.418	0.141	–1.087	0.281
Q17	Disappointed	300	1.617	1.471	0.334	0.141	–1.326	0.281
Q18	Angry	300	1.613	1.462	0.402	0.141	–1.205	0.281
Q19	Energetic	300	1.960	1.430	0.009	0.141	–1.292	0.281
Q20	Happy	300	1.873	1.423	0.057	0.141	–1.311	0.281
Q21	Anxious	300	1.760	1.457	0.221	0.141	–1.275	0.281
Q22	Dejected	300	1.460	1.557	0.560	0.141	–1.240	0.281

The mean estimates of the items ranged from 1.443 (Q2, “*upset*”) to 2.090 (Q10, “*joyful*”). No missing data were recorded on any of the items. Though these mean values appear quite low, other studies (e.g., Vast et al., 2010; Biscaia et al., 2012) also reported relatively low mean values on all or some of the sub-domains. The skewness values ranged between −0.014 to 0.560 which was within the acceptable range of-2 to + 2. The kurtosis values ranged from -0.832 to 1.326, which was also within the acceptable range of -7 to + 7 (Bryne, 2010).

### 5-factor confirmatory MIRT model

Table 2 presents the results concerning the confirmatory MIRT model.

**TABLE 2:** Discrimination and threshold parameters for the 5-factor confirmatory MIRT model.

Item	a_1_	a_2_	a_3_	a_4_	a_5_	d_1_	d_2_	d_3_	d_4_
Q1	0.724					1.204	–0.04	–1.060	–1.786
Q6	1.391					1.525	0.113	–1.137	–2.778
Q11	0.938					1.076	0.456	–0.663	–1.945
Q16	0.421					0.985	–0.01	–0.951	–1.718
Q21	0.318					0.958	0.23	–0.777	–1.496
Q2		1.388				1.113	–0.052	–0.644	–2.914
Q7		1.303				1.401	0.495	–0.483	–1.226
Q12		3.659				1.235	0.328	–0.647	–1.961
Q17		0.607				1.530	0.514	–0.625	–1.914
Q22		4.930				0.778	–0.038	–0.732	–1.358
Q4			1.454			1.221	0.533	–0.702	–2.576
Q9			0.702			1.585	0.447	–0.796	–1.721
Q14			0.747			0.880	–0.015	–1.082	–1.637
Q18			1.03			1.249	0.386	–0.531	–1.727
Q3				1.716		1.363	0.048	–0.647	–1.854
Q8				0.171		1.778	0.632	–0.335	–1.381
Q13				0.290		1.440	0.637	–0.711	–1.593
Q19				3.079		1.530	0.514	–0.625	–1.914
Q5					1.073	2.081	0.970	–0.564	–2.081
Q10					0.96	1.242	0.626	–0.622	–1.795
Q15					1.361	0.898	0.041	–0.605	–1.638
Q20					0.783	1.249	0.386	–0.531	–1.727

a_1_, Anxiety; a_2_, Rejection; a_3_, Anger; a_4_, Excitement; a_5_, Happiness; *d*, item easiness.

The results from the confirmatory MIRT model based on 5-factors with 22 items revealed that whereas some items discriminated very well, others had very low discrimination parameters. For factor 1, that is the Anxiety dimension, the discrimination parameters ranged from 0.318 (Q21, *“anxious*”) to 1.391 (Q6, *“tense”*). Factor 2, the Rejection dimension, had a discrimination indices from 0.607 (Q17, “*disappointed*”) to 3.659 (Q12, “*unhappy*”). The other factors had the following discrimination indices: Anger dimension from 0.702 (Q9, “*furious*”) to 1.454 (Q4, “*irritated*”); Excitement dimension from 0.171 (Q8, “*excited*”) to 3.079 (Q19, “*energetic*”); and happiness dimension from 0.783 (Q20, “*happy*”) to 1.361 (Q5, “*please*”). The threshold parameters found no disordered threshold (see Table 2). However, there were a few abnormalities in the category functioning based on the recommended threshold step as provided by Wolfe and Smith (2007). According to Wolf, this threshold step should not be less than 0.81. For item 1 (Q1), the threshold 3 (d_3_) between the responses of “*moderately*” and “*quite a bit*” was −1.060 whereas that of “*quite a bit*” and “*extremely*” was −1.786, leading to a threshold step of 0.726, indicating that the “*quite a bit*” category appeared problematic. Similar concerns were found with items 14 (threshold step of 0.555) and 16 (threshold step of 0.767).

### Bifactor MIRT model

The outcome of the analysis concerning the bifactor MIRT model is presented in Table 3.

**TABLE 3:** Discrimination and threshold parameters for the bifactor MIRT model.

Item	G	a_1_	a_2_	a_3_	a_4_	a_5_	d_1_	d_2_	d_3_	d_4_
Q1	0.843	0.050					1.238	–0.061	–1.153	–1.909
Q6	0.585	0.522					1.281	0.027	–1.111	–2.492
Q11	1.070	1.938					2.301	0.582	–1.347	–2.658
Q16	1.556	0.773					1.232	–0.053	–1.578	–2.351
Q21	0.426	0.181					0.968	0.232	–0.807	–1.554
Q2	1.315		0.301				0.807	–0.484	–1.141	–3.484
Q7	0.996		0.706				0.877	0.048	–0.925	–2.192
Q12	0.923		0.762				0.716	0.156	–1.15	–2.319
Q17	1.813		1.087				1.134	–0.2	–1.243	–2.857
Q22	1.111		0.570				0.311	–0.522	–1.258	–1.892
Q4	0.893			0.524			1.025	0.354	–0.87	–2.524
Q9	0.823			0.171			1.375	0.407	–0.623	–1.387
Q14	0.967			0.844			1.274	0.244	–0.857	–2.265
Q18	2.063			2.716			1.759	–0.282	–2.218	–3.667
Q3	0.690				0.615		1.225	0.393	–0.721	–1.776
Q8	0.547				0.073		1.461	0.454	–0.429	–1.317
Q13	0.574				0.449		1.139	0.452	–0.777	–2.108
Q19	0.132				0.041		1.234	0.448	–0.494	–1.413
Q5	0.032					0.946	2.082	1.021	–0.56	–2.181
Q10	0.790					0.669	1.872	0.678	–0.368	–1.474
Q15	0.682					1.273	1.569	0.688	–0.865	–1.803
Q20	0.016					0.803	1.276	0.391	–0.563	–1.79

a_1_, Anxiety; a_2_, Rejection; a_3_, Anger; a_4_, Excitement; a_5_, Happiness; *d*, item easiness; G, general factor.

The results from the bifactor MIRT model revealed that the majority of the items showed a higher discrimination index on the general factor (sports emotion) as compared to the individual factors. For example, item 1 had a discrimination index of 0.843 on the general factor and 0.050 on the Anxiety sub-domain, suggesting that item 1 offers more information to the general factor than the group factor (i.e., Anxiety domain). Similarly, item 17 had a discrimination parameter of 1.813 on the general factor and 1.087 on the Rejection subscale. There were a few instances where some items had high discrimination indices on the group factor as compared to the general factor. Item 18, for instance, had a discrimination parameter of 2.063 on the general factor and 2.716 on the Anger dimension. Other items like Q11, Q5, Q15, and Q20 had similar result patterns.

### Confirmatory vs. bifactor MIRT models

Comparing the confirmatory and bifactor MIRT models, it was found that the bifactor MIRT model showed significant improvement over the confirmatory MIRT model. In the confirmatory model, for example, item 22 had a discrimination index of 4.930, which may perhaps indicate that the item is a good indicator for the Rejection domain. Nevertheless, this item has a discrimination parameter of 1.111 on the general construct and 0.570 on the Anxiety subscale. This suggests that this item (under the bifactor model) is approximately two times more informative on the general construct as compared to the specific dimension.

As shown in Table 4, two models were fitted for the 5-factor first-order model (CFI = 0.898, TLI = 0.820, SRMSR = 0.037, RMSEA = 0.024) and the bifactor model (CFI = 0.943, TLI = 0.923, SRMSR = 0.036, RMSEA = 0.023). The results showed that the bifactor model showed an improvement over the 5-factor model. Further, a significant difference was found between the two models with regard to the fitting information of each model, *p* < 0.001. That all the model fit indicators were in favor of the bifactor model, with the model showing the least values of M2, AIC, SABIC, BIC, and LL. Thus, the bifactor model was supported indicating that a within-item multidimensional theory (an item can measure more than two latent traits) framework is suitable in terms of the utility of the SEQ in athletes in Africa.

**TABLE 4:** Model fitting indicators for confirmatory and bifactor MIRT models.

Model fit indicators	5-factor CFA (first-order)	Bifactor MIRT
CFI	0.898	0.943
TLI	0.820	0.923
SRMSR	0.037	0.036
RMSEA	0.024	0.023
M2	938.79	777.57
Akaike information criterion	20184.16	19699.01
Bayesian information criterion	20673.06	20328.65
Sample adjusted BIC	20254.44	19789.51
Log-likelihood (LL)	-9960.082	-9679.505
	

Δ X^2^ (38) = 561.154, *p* < 0.001.

### Relevant indices for the bifactor MIRT

Other relevant bifactor indicators were computed for greater insight in terms of understanding the latent structure of the SEQ. Table 5 presents the output of the analysis.

**TABLE 5:** Bifactor indices for the specified model.

Domains	ECV (S&E)	ECV (NEW)	ω/ω_s_	ω _H_/ω _HS_	Relative omega	H	FD
General factor	0.740	0.740	0.946	0.902	0.954	0.946	0.969
Dejection	0.059	0.250	0.817	0.048	0.059	0.532	0.875
Anxiety	0.044	0.152	0.905	0.128	0.142	0.364	0.753
Anger	0.059	0.338	0.773	0.198	0.257	0.548	0.966
Happiness	0.022	0.160	0.709	0.076	0.108	0.229	0.549
Excitement	0.075	0.474	0.762	0.359	0.471	0.533	0.775

Total Omega (ω), Subscale Omega (ω_s_), Hierarchical Omega (ω_H_), Hierarchical Omega for subscales (ω_HS_), Percent of Uncontaminated Correlation (PUC) = 0.835.

The output of the bifactor indices analysis showed that about 74% of the common variances can be attributed to the general emotion factor (Table 5). In contrast, all the sub-domains of the SEQ exhibited weak ECV estimates (i.e., ECV_S values ranged from 0.022 to 0.075; ECV_NEW values were between 0.152 and 0.474) compared to all explained variances of all the items, whether items that loaded or not were included. The omega estimates across the general and specific factors also support the values from the ECV measures indicating that the total scores from the SEQ can essentially be considered unidimensional. Comparing the *H* indices for the general and specific factors revealed that the set of 22 items represented the general emotion factor. Additionally, values from both the PUC and ECV supported treating the SEQ as if it was a unidimensional scale.

## Discussion

This study examined the factor structure of SEQ among athletes from West Africa using 5-factor CFA and bifactor MIRT models. The 5-factor confirmatory MIRT revealed varying levels of discrimination among the SEQ items. Four items across three dimensions had low discriminating powers: Q16 and Q21 under the anxiety subscale, and Q8 and Q13 under the excitement subscale. These indices suggest that these items could not highly discriminate among athletes in terms of their intensities of anxiety, rejection, and excitement (Reckase and McKinley, 1991). Largely, the thresholds for the 5-factor confirmatory MIRT indicated that the probability of endorsing an item increased with increasing latent traits for every combination of the domain. This implies that with an increasing level of the latent trait on any of the sub-domains of SEQ, athletes are more likely to endorse higher response categories such as “*quite a bit*” and “*extremely*” than the lower response categories such as the “*not at all*” and “*a little*.” This means that the items were easy for athletes high on the latent trait for any of the domains to endorse high response categories such as “*quite a bit*” and “*extremely*.” Examination of the threshold steps indicates some items (e.g., Q1, Q14, and Q16) had slight problems with the category functions for “*moderately*” and “*quite a bit*” response options. The fourth category (*quite a bit*) for the aforementioned items appeared problematic, as their threshold steps were less than 0.81 (Wolfe and Smith, 2007). There is the possibility that the athletes did not discriminate between the “*quite a bit*” and other adjacent response options, hence athletes with varying levels of emotions endorsed the same response option, *quite a bit*’. Following this, possibly, for those items, the “*quite a bit*” and “*extremely*” response categories could be merged into one. Alternatively, responses for “*moderately*” and “*quite a bit*” could be collapsed as one to reduce the threshold gap.

Further, results from the bifactor MIRT model identified four items on the general factor (Q21, Q19, Q5, and Q20); and seven items on the group factors—anxiety (Q1, Q21), rejection (Q2), anger (Q9), excitement (Q8, Q13, Q19)—poorly discriminating among the levels of the construct. This suggests that more items on the group factor than the general factor (sport emotion) could not discriminate highly among the levels of emotion construct of the athletes (Reckase and McKinley, 1991). Nevertheless, the general factor saw more items with high discrimination abilities than the group factor. Besides, other indices from the bifactor model supported the fact that the general factor explained more variances of the construct than the group factors. This signifies that the general factor brought about differences in athletes’ sport emotions better than the group factor, confirming a unidimensional model. Undoubtedly, the unidimensional structure appears to explain the underlying latent structure of sports emotion better than the multidimensional structure. In view of that, it can be argued that a unidimensional emotion scale relative to the multidimensional scale could provide more information on varying levels of athletes’ sport emotions and also distinguish precisely among the intensities of their sport emotions. In confirmation, the model fit indices for all the information criteria for the bifactor model were better than the confirmatory model, suggesting that an item could be measuring more than a single latent trait.

More specifically, considering items Q3 “exhilarated,” Q8 “excitement,” and Q13 “enthusiastic” for the excitement dimension and items Q5 “pleased,” Q10 “joyful,” Q15 “cheerful,” and Q20 “happy” for the happiness dimension, presents an interesting view of SEQ utility and functioning. These proxies, semantically, appear to be describing the same state, but with different words. For instance, how different is it when an athlete is excited and concurrently happy? Put differently, how different is an excited athlete from a happy athlete? There is difficulty in drawing a distinction between these items, and this may suggest the redundancy of items or items belonging to a single subscale. Predictably, all the items on the excitement dimension discriminated better on the general factor (sports emotion) than on its original dimension (i.e., excitement), suggesting a possible dissolution of the excitement dimension since its items do not provide maximum information on athletes’ emotions as required. It is also not surprising that all the items accurately represented the general factor compared to their representation of their respective sub-domains. Supporting this, Latinjak et al. (2013) found the proxies for excitement and happiness constructs as lacking distinction. Similarly, Vast et al. (2010) also found a strong positive relationship between the indicators for excitement and happiness dimensions, indicating a strong association between them and possibly contributing the same or similar empirical information to the construct being measured. Further examination of the three other dimensions of SEQ, namely anxiety, rejection, and anger suggests a similar view as in the case of excitement and happiness. Superficially, indicators for classifying an anxious person is reflected in proxies for rejection and anger. Likewise, people who are angry exhibit behaviors related to anxiety and rejection. Of course, for the anxiety and anger dimensions, all their items saw high discrimination powers on the general factor (sports emotion) except for one item in each case. Similarly, all the items on the rejection dimension discriminated better on the general factor (sports emotion) than on itself. All of these confirm a unidimensional model.

Summarily, evidence from this study provides minimal support for the usual 5-factor structure of the SEQ (Jones et al., 2005; Vaez-Mosavi and Eshghi, 2020; Gomes et al., 2021), rather, a unidimensional model is largely supported. An implication is that a single latent trait best explains athletes’ emotions. Contrary to the findings of this study, some studies have found 3-factor (Latinjak et al., 2013; Wetzel et al., 2020), 4-factor (Bayköse and Şakar, 2018), and 5-factor structures but with partial support for the structure of the original version (Arnold and Fletcher, 2015). Admittedly, looking at how sports emotion was measured in this study and in some other studies, the variations in the results are not surprising, acknowledging that emotions can be either a trait- or state-like construct. As a trait, sports emotion is durable and relatively stable over time and across situations, however, as a state, it represents reactive feelings to specific situations at particular time periods (Ye et al., 2020). In this study, for instance, emotion was measured as a state-like construct where participants were asked to indicate how they were feeling “right now, at this moment, regarding the upcoming competition” within the competitive setting in the week prior to the actual competition, which is similar to that of Jones et al. (2005). Latinjak et al. (2013), also used the preamble “usually in a situation, I feel …..” In the case of Latinjak et al. participants may be reporting either their previous or current reaction to sport and with this, the cause of the emotional disposition might not be known. From the perspective of trait and state, depending on the context, nature of the situation and its importance, varied levels of emotions could be exhibited by different athletes, therefore, it is not surprising the findings of this study contradict those of previous studies.

Considering the nature and the directions of the items of the SEQ, a unidimensional factor structure is proposed to be tested by further studies. These may generally look at emotions as a general factor, where excitement and happiness, anxiety, rejection, and anger sub-dimensions are uncorrelated. This research contributes to the cross-cultural discussion on the utility and use of the SEQ through a more intensive approach to calibration. Most important, the study starts discussions on the structural validity of the SEQ within the African setting based on the notion that emotions represent diverse societies and socially constructed values (Hagan, 2021).

### Practical implications

The outcome of this research contributes to the literature on the sports psychology-related field. Based on these findings, sport psychologists should be guided regarding the use of SEQ for future research; particularly, the instrument should be used as if it is a unidimensional scale. It must be emphasized that users of the SEQ should refrain from treating the specific domains of the SEQ as distinct for analysis purposes. This idea stems from the evidence that the items did not contribute meaningfully to their sub-domain as they did for the general measure of emotions. Moreover, coaches and psychologists of various sporting disciplines can use the instrument to accurately scale athletes into those experiencing varied forms of emotions for appropriate interventions to be offered to them. This is relevant as previous studies have revealed a strong link between emotions and sports performance (Lazarus, 2000; Jones and Uphill, 2011). Further, scholars who wish to ascertain the efficacy of emotion-based interventions or programs can utilize the SEQ to precisely scale subjects based on their emotional disposition.

### Limitations

The study was conducted using participants in the African setting and thus, the representativeness and generalization of the findings of this research are limited to a population outside this boundary. Also, there are several recommendations in the measurement literature stating that a sample size of more than 500 is warranted to ensure precision in parameter estimation for multidimensional models. This study, on the other hand, used 300 cases for the estimation. Thus, statistical power might be affected. Future studies should replicate this study using large sample size and issues of invariance across relevant variables should also be considered in future research. Another key limitation of the research is the non-availability of software packages used to perform exploratory bifactor IRT analyses. As a result, the investigators did not follow up on the findings to test the unidimensionality of the SEQ within this framework.

## Conclusion

The findings from our study provide evidence against the dimensionality of the 5-factor SEQ and questioned the utility and utilization of the sub-dimensions of the SEQ. There were traces of evidence showing that most of the items on the SEQ were more discriminating proxies of the general factor and contributed significantly to the measurement of the general emotion variable than any of the five domains. Though this study does not take a strong position on the removal of the sub-domains of the SEQ, it is strongly advised that participants’ scores on the instrument should be interpreted based on the general score paying little attention to the specific sub-scales, especially when performing inferential analyses.

## Data availability statement

The raw data supporting the conclusions of this article will be made available by the authors, without undue reservation.

## Ethics statement

All ethical procedures were in line with the Institutional Review Board (IRB) at Bielefeld University and adhered to the ethical standards of the sixth revision of the Declaration of Helsinki. The participants provided their written informed consent to participate in this study.

## Author contributions

JH and FQ conceived the idea. FQ performed the analysis. JH, FQ, FA, MS-S, and TS prepared the initial draft of the manuscript. All authors thoroughly revised and approved the final version of the manuscript.

## Footnotes

1
https://cran.r-project.org/web/packages/mirt/index.html
